# Acetyl-L-carnitine (ALCAR) to enhance nerve regeneration in carpal tunnel syndrome: study protocol for a randomized, placebo-controlled trial

**DOI:** 10.1186/s13063-016-1324-2

**Published:** 2016-04-14

**Authors:** Matthew W. T. Curran, Jaret Olson, Michael Morhart, Dory Sample, K. Ming Chan

**Affiliations:** Walter Mackenzie Centre, University of Alberta Hospital, 8440 112 St, Edmonton, AB T6G 2B7 Canada; 2D3 Walter Mackenzie Centre, University of Alberta, 8440 112 St, Edmonton, AB T6G 2B7 Canada; 303 Coronation Plaza East Tower, Edmonton, AB T5M 3Z7 Canada; Women and Children’s Health Research Institute, 4-081 Edmonton Clinic Health Academy, University of Alberta, 11405 – 87 Ave NW, Edmonton, AB T6G 1C9 Canada; 5-005 Katz Group Centre, University of Alberta, Edmonton, AB T6G 2E1 Canada

**Keywords:** Carpal tunnel syndrome, Peripheral nerve injury, Acetyl-L-carnitine, Motor unit number estimation, Nerve regeneration

## Abstract

**Background:**

Carpal tunnel syndrome (CTS) is the most common form of peripheral nerve injury, affecting approximately 3 % of the population. While surgery is effective in mild and moderate cases, nerve and functional recovery are often not complete in severe cases. Therefore, there is a need for adjuvant methods to improve nerve regeneration in those cases. Acetyl-L-carnitine (ALCAR) is involved in lipid transport, vital for mitochondrial function. Although it has been shown to be effective in various forms of neuropathies, it has not been used in traumatic or compressive peripheral nerve injury.

**Methods:**

In this pilot study we will utilize a double-blind, randomized, placebo-controlled design. Inclusion criteria will include adult patients with severe CTS. This will be confirmed by nerve conduction studies and motor unit number estimation (MUNE). Only those with severe motor unit loss in the thenar muscles (2 standard deviations [SD] below the mean for the age group) will be included. Eligible patients will be randomized to receive 3,000 mg/day of ALCAR orally or placebo following carpal tunnel release surgery for 2 months. The primary outcome will be MUNE with supplementary secondary outcome measures that include: 1) two-point discrimination; 2) Semmes-Weinstein monofilaments for pressure sensitivity; 3) cold and pain threshold for small fiber function; 4) Boston self-assessment Carpal Tunnel Questionnaire and 5) Disabilities of the Arm, Shoulder and Hand (DASH) questionnaire for symptom severity; and 6) Purdue Pegboard Test for hand functional performance. To follow post treatment recovery and monitor safety, patients will be seen at 3 months, 6 months and 1 year. The outcome measures will be analyzed using two-way ANOVA, with treatment assignment and time points being the independent factors. If significant associations are detected, a post hoc analysis will be completed. We aim to recruit ten patients into each of the two groups. Data from this pilot will provide the basis for power calculation for a full-scale trial.

**Discussion:**

ALCAR is a physiologic peptide crucial for fatty acid transport. ALCAR has been shown to be effective in neuroprotection in the central nervous system and increase peripheral nerve regeneration. This has been applied clinically to various systemic peripheral neuropathies including diabetic neuropathy, antiretroviral toxic neuropathy, and chemotherapy-induced peripheral neuropathy. While animal evidence exists for the benefit of ALCAR in compression neuropathy, there have been no human studies to date. This trial will represent the first use of ALCAR in peripheral nerve injury/compression neuropathy.

**Trial registration:**

NCT02141035; 20 April 2015

## Background

Carpal tunnel syndrome (CTS) is the most common peripheral nerve injury. Based on the clinical assessment of 3,000 randomly chosen individuals in Sweden, the prevalence of confirmed CTS was almost 3 % in the general population [1]. Of those, 25 % had severe symptoms and nerve injury that could not be sufficiently managed through conservative means [2]. The large number of afflicted individuals poses a major healthcare cost and social economic burden [3]. Indeed, it is estimated that up to half a million patients undergo surgery for CTS each year in the United States with an associated healthcare cost that exceeds $2 billion [4]. Therefore, much effort has been spent on attempts to improve treatment outcomes. Although conservative measures such as wrist splinting can be effective in milder cases, surgery is needed to relieve pressure on the median nerve when conservative measures fail. However, even with surgery, the outcome in more severe cases in which substantial axonal degeneration has already occurred remains poor [5]. One major reason is that even though injured nerve fibers have an inherent ability to regenerate, the regeneration proceeds very slowly with rates of only 1 mm/day in ideal circumstances [6, 7]. The regenerative capacity of axons and the Schwann cell (SC) growth support decline with time and distance from injury [8, 9].

To improve the speed and extent of nerve regeneration, novel treatment strategies are being explored. While decompression surgery is useful in alleviating further mechanical injury to the median nerve, it does little to influence the underlying molecular mechanisms of peripheral nerve regeneration. Although a number of potential therapeutic agents including FK506 [10], geldanamycin [11] and fibroblast growth factor [12] have been studied, their safety and tolerability for human application have challenges. In contrast, acetyl-L-carnitine (ALCAR) has an excellent safety track record for human use. It is a naturally occurring molecule that acts as an acetyl donor group that is critical for lipid transport and the maintaining of mitochondrial function. In vitro studies showed that ALCAR promoted cerebellar neuronal maturation and increased peripheral nerve regeneration in animal studies [13]. These findings prompted the evaluation of ALCAR in various neuropathies to improve nerve regeneration. Clinical conditions studied include antiretroviral toxic neuropathy [14], diabetic neuropathy [15] and chemotherapy-induced neuropathy [16]. Those studies showed that patients treated with ALCAR have less pain and better motor and sensory function. While this data is encouraging, the use of ALCAR in patients with compressive neuropathies remains largely untested. In an animal study, Kotil et al. found histopathological evidence of improvement when ALCAR was administered on its own or with concomitant decompression surgery [17]. The only human study to date that includes compression neuropathies was primarily designed to examine the tolerability of ALCAR [18]. However, evidence of nerve regeneration was not a primary objective of that study.

### Objective

The objective of the trial is to determine the efficacy of ALCAR on nerve regeneration in patients with severe CTS.

### Hypothesis

ALCAR will significantly increase motor axonal regeneration, improve sensory function and more rapidly resolve CTS symptoms.

## Methods

### Basic trial design

This double-blinded, placebo-controlled trial has received ethics approval from the University of Alberta Health Ethics Research Board (Pro00045538). The study protocol has been reviewed by Health Canada, and a No Objection Letter (NOL) has been issued for the use of ALCAR. Subjects will be recruited from plastic surgery clinics at the University of Alberta Hospital and Royal Alexandra Hospital as well as the electromyography (EMG) clinic at the Glenrose Rehabilitation Hospital.

### Subjects

All patients referred to either the plastic surgery clinics or the EMG lab will be eligible to be screened. Inclusion into the study will be based upon the criteria listed in Table 1. Patients meeting eligibility criteria will be recruited and informed consent will be obtained. They will then undergo motor unit number estimation (MUNE) to quantify the severity of motor axonal loss. Only those with motor axonal loss of > 2 SD below the mean for the age group will be included in the study.

**Table 1:** Inclusion and exclusion criteria for patient selection

Inclusion criteria	Exclusion criteria
1) Age > 18 years	1) Motor unit loss in the median nerve less than 2 standard deviations below the mean for the age group
2) One or more of the following symptoms of CTS	2) Presence of other neurologic conditions
a) Numbness and paresthesias in the median nerve distributions	3) Previous carpal tunnel release
b) Precipitation of those symptoms by repetitive motions that are relieved by rubbing and/or shaking	4) Cognitive impairment that renders the patient incapable of providing consent
c) Nocturnal awakening of the above symptoms	5) History of seizures
d) Weakness of thumb abduction and thenar atrophy	6) Kidney disease/renal impairment
	7) Sensitivity to any of the drug components of the formulation or are
	8) Pregnant/breast feeding
	9) For women of child-bearing potential, those that are not willing to use adequate contraceptive prevention methods for at least 30 days after the last dose of the medication.

### Randomization

After enrollment, patients will be randomized to placebo or ALCAR. A randomization sequence will be generated electronically and kept by a statistician not directly involved in patient care. Randomization will be kept on a computer under password encryption. The randomization code will only be broken if a patient experiences a severe adverse event. At the end of the study the statistician will provide the research team with the randomization codes.

### Blinding

The study will be administered in a double-blind manner. The randomization sequence will be kept confidential and only accessible to authorized personnel. The pharmacy will distribute identical pills in bottles labeled with A or B. The research team will be unaware of which contains the study drug or placebo. This will be maintained until the final follow-up has occurred, at which point the research team will be unblinded.

### Primary outcome

The primary endpoint will be motor axon reinnervation in the thenar muscles. This will be done using MUNE, a non-invasive electrophysiological technique. Initially introduced in the 1970s [19, 20], multipoint stimulation, the most commonly used MUNE technique, has been shown to be a reliable test for measuring motor axon reinnervation [5]. Apart from providing an objective assessment of large myelinated nerve fibers, MUNE will also serve as a screening tool for eligibility for the trial. To quantify treatment response, MUNE will be repeated at 3, 6 and 12 months post-operatively.

### Secondary outcomes

Secondary outcomes of the study focus on sensory function restoration and functional outcomes. Measures will include:

1. Two-point discrimination (Aβ fibers) using Dellon-MacKinnon Disk
2. Pressure sensitivity (Aβ fibers) using Semmes-Weinstein monofilament
3. Cold threshold (Aδ fibers) and pain threshold (C fibers) using CASE IV quantitative sensory testing (QST) equipment
4. Boston CTS Questionnaire to monitor symptom severity
5. Disabilities of the Arm, Shoulder and Hand (DASH) questionnaire for functional outcomes
6. Moberg Pick-up Test to measure hand dexterity and functional performance

All outcome measures will be monitored at baseline, 3 months, 6 months and 12 months. Sensory testing of peripheral nerves provides more of a challenge, given the multiple fiber types that provide sensory feedback. The above list of tests is designed to capture the full range of sensory fiber functions. A major advantage of QST is that it provides an objective means of measuring the function of different classes of nerve fibers with good reliability. The DASH and the Boston questionnaires used to evaluate symptom severity are both well validated in CTS [21]. Finally, to objectively measure hand functional performance, the Moberg Pick-up Test is a validated tool for CTS with good reliability [22]. All primary and secondary outcome measures will be performed by the same investigators (MC, KMC). To verify the inter-rater reliability of these tests, a subset of patients will undergo separate assessment by two investigators independently in a blinded manner.

### Interventions

Patients will be assigned to either the placebo or ALCAR arm of the study. Both placebo and ALCAR will be fabricated by the same pharmaceutical company (Sigma-Tau, Rome, Italy). They are identical in appearance and taste. Upon arrival, the tablets will be packaged by the pharmacy into identical boxes labeled with a random sequence code. Both groups will undergo open carpal tunnel release without epineurolysis performed by experienced hand surgeons (JO, MM). Post-operatively, patients will take the medications for 2 months. Patients randomized to the ALCAR arm will take 3,000 mg orally divided in three times per day dosing, while the control group will take a matched placebo. The dosing schedule for the study drug was chosen based on previous work by Bianchi et al. (2005) that showed good tolerability and efficacy [23].

### Sample size

Initially, a pilot study of 20 patients will be carried out. Evaluation of the effect size will be based on the primary outcome MUNE. This will provide the necessary data for the sample size calculation for a fully powered large-scale study.

### Statistical analysis

Demographic data will be compiled and analyzed for differences using a Student’s *t*-test and Fisher’s exact test. The primary and secondary outcome measures will be analyzed using two-way ANOVA with the treatment groups and time points being the independent factors. If significant associations are detected, a post hoc analysis will be done for pairwise comparisons. Due to the exploratory nature of the study, sample size calculations and formal analysis of missing data will not be undertaken.

### Safety and potential risks

To monitor any potential adverse events, rigorous safety measures have been put in place to ensure patient safety. Initial baseline testing will include:

1. Physical examination
2. Vitals
3. EKG
4. Urine pregnancy tests
5. Complete blood count with differential, electrolytes (Na, K, Cl, CO_2_), creatinine, urea, alanine aminotransferase, aspartate aminotransferase, alkaline phosphatase and bilirubin
6. Serum ALCAR

Vital signs (blood pressure, temperature, pulse, respiratory rate, oxygen saturation) will be recorded at every visit. Safety lab tests including blood chemistries will be done at screening, and periodically during follow-up visits. In addition, an electrocardiogram will be done at screening. The serum ALCAR level will also be monitored. In healthy subjects, the serum ALCAR levels had a range of 10–70 μmol/L with a mean of 39 μmol/L. In a large study of over 333 patients with diabetic neuropathy taking 3 g of ALCAR/day (the same dose used in this study), the rise in serum level was only around 50 % compared to baseline. Since no sign of ALCAR toxicity was observed in any of the subjects, we anticipate that the chances of toxic effects in this study will be very small.

All adverse events will be documented at follow-up visits. Further laboratory investigations will be done depending on the nature and severity of the adverse events reported.

### Trial management

The trial will be managed by a trial steering committee in accordance with Good Clinical Practice guidelines. The principle investigator, K. Ming Chan (KMC), will be primarily responsible for the overall trial functioning with assistance from Matthew Curran (MC). The administration of the study drug will be completed through the Research Pharmacy Office located at the Kaye Edmonton Clinic. Adverse events will be tracked and managed by a Drug Safety Monitoring Board. The trial has been registered with Clinicaltrials.gov (CT02141035)*.*

### Research team

Our group, as part of the Western Canada Regeneration Initiative, has a substantial track record in developing novel treatments for patients with peripheral nerve injury. KMC is an experienced clinician scientist in physical medicine and rehabilitation who will be responsible for evaluating the treatment outcomes and for overseeing the project. MC, a resident in the plastic surgery department, will be responsible for recruitment of patients, evaluating treatment outcomes and safety, data analysis and manuscript preparation. Jaret Olson (JO) and Michael Morhart (MM) are plastic surgeons who will be responsible for patient recruitment and performing the carpal tunnel release surgery. All investigators were involved in the study design and will continue to have a role in result analysis and writing of the manuscript.

### Ethics

The study has ethical approval from the Health Research Ethics Board (Pro00045538) at the University of Alberta. The trial will be conducted in keeping with the principles of Good Clinical Practice.

## Discussion

Acetyl-L-carnitine is a peptide natural product that plays a crucial role in fatty acid metabolism, acyl group transfer and transport into mitochondria. It has been shown to be beneficial in a number of disease states including oligospermia [24], hepatic encephalopathy [25] and dysthymia [26]. It has also been shown to have a positive impact on central and peripheral nervous system diseases. It can reverse signs of aging in rat brains [27]. In peripheral neuropathies it is thought that ALCAR enhances nerve growth factor receptor binding, allowing for neurite outgrowth through the ERK pathway [13, 28]. This effect has been demonstrated in diabetic neuropathy [15], chemotherapy-induced peripheral neuropathy [16] and antiretroviral toxic neuropathy [14]. ALCAR is very well tolerated. Large trials have shown minimal side effects. Its practicality is further enhanced by the fact that the drug is active in oral preparation.

### Strengths

Vigorous measures have been built into the study design. To prevent potential bias, we utilize a double-blinded, placebo-controlled design. To further reinforce this, data entry and review will be handled by a monitoring committee that will be working at arm’s length from the investigators who are directly interacting with the study subjects. Surgery will be performed by two experienced surgeons using a standardized technique for carpal tunnel release. To measure the effects of the therapeutic agent on nerve regeneration, we will be using MUNE as the primary outcome measure. This provides a physiological measure that can be directly compared with the data from animal studies. In addition, the protocol will also incorporate tests to provide comprehensive information on the full range of sensory nerve fibers.

### Limitations

A potential limitation of our study is generalizability of the results. Since we will be deliberately focusing on only CTS patients with severe motor axonal loss, the results may not be applicable to those with mild and moderate disease. However, since severe carpal tunnel patients have the lowest potential for recovery even with surgery, it would be important to target this population. A second limitation of the study is that it is likely underpowered. However, since no prior studies on the effect size and variability of ALCAR in compressive neuropathy are available, data from this pilot study will be very useful in designing a full-scale study. The multiple comparisons would also reduce the statistical power of the study. Since this is meant to serve as an exploratory study, these considerations are not of primary concern.

Here we present a phase II randomized control trial design to determine the role of ALCAR in peripheral nerve regeneration in CTS. This is the first human study that looks at the efficacy of ALCAR in patients with compressive neuropathy. If effective, ALCAR will represent an important adjuvant therapy for patients with CTS. By improving motor and sensory recovery, patients could potentially return to their work and daily routines more quickly.

### Trial status

Currently recruiting NCT02141035.

## Abbreviations

ALCAR: acetyl-L-carnitine
ANOVA: analysis of variance
CTS: carpal tunnel syndrome
EMG: electromyography
JO: Jaret Olson
KMC: K. Ming Chan
MC: Matthew Curran
MM: Michael Morhart
NOL: No Objection Letter
MUNE: motor unit number estimation
QST: quantitative sensory testing
SC: Schwann cell
SD: standard deviation
